# Serum cholinesterase as a new nutritional indicator for predicting weaning failure in patients

**DOI:** 10.3389/fmed.2023.1175089

**Published:** 2023-07-12

**Authors:** Jiaping Liu, Tianyu Shao, Hanwen Chen, Chenyang Ma, Xiaohui Lu, Xiaoming Yang, Kang Song, Lu Wang, Shu Lei, Dafen Wang

**Affiliations:** ^1^The First School of Clinical Medicine, Zhejiang Chinese Medical University, Hangzhou, China; ^2^Department of Oncology, Guang' Anmen Hospital, China Academy of Chinese Medical Sciences, Beijing, China; ^3^Department of Traditional Chinese Medicine, The Second People’s Hospital of Xiaoshan District, Hangzhou, China; ^4^The First Affiliated Hospital of Zhejiang Chinese Medical University (Zhejiang Provincial Hospital of Chinese Medicine), Hangzhou, China

**Keywords:** serum cholinesterase, weaning, invasive mechanical ventilation, nutrition, autonomic breathing test

## Abstract

**Aim:**

The objective of this study is to examine the correlation between patient serum cholinesterase (SCHE) concentration and weaning failure in the context of invasive mechanical ventilation (IMV), as well as to identify predictors of ventilator weaning failure. Additionally, this study investigates the potential relationship between SCHE and nutritional risk for developing more effective weaning strategies.

**Method:**

A retrospective observational study was conducted. The sample was collected from 227 patients with IMV over 48 h who underwent SBT before weaning. Relevant experimental samples and data collection were analyzed at the time of patient admission and before the initiation of the SBT. The correlation between SCHE and weaning failure was determined by multifactorial logistic regression and propensity matching scores.

**Results:**

Weaning was successful in 127 patients and failed in 100 patients. Depending on the difficulty of weaning, 55 of these patients had difficulty in weaning and 45 had long-term weaning. In the crude cohort, experimental data collected on the day of SBT showed that SCHE concentrations were higher in patients with successful weaning than in those with failed weaning (4,514 u/l vs. 3,190 u/l *p* < 0.01). The critical value for predicting weaning failure was SCHE 3,228 u/l (*p* < 0.01). Ventilator weaning failure was predicted by multifactorial logistic regression analysis of SCHE, heart rate, and PaO_2_ before SBT, with SCHE predicting ventilator weaning failure (AUC 0.714; 95% CI 0.647–0.782) better than heart rate (AUC 0.618; 95% CI 0.545–0.690), PaO_2_ (AUC 0.59; 95% CI 0.515–0.664). After propensity-matched scores, SCHE remained an independent predictor of weaning failure (*p* = 0.05). And the SCHE concentration was strongly correlated with the patient’s weaning difficulties (*p* < 0.01). The Nutrition Risk in Critically Ill (NUTRIC) score was also significantly correlated with SCHE according to Spearman’s correlation analysis (*p* < 0.01).

**Conclusion:**

Our study revealed that the patients who experienced weaning failure exhibited lower SCHE values compared to those who successfully underwent weaning. Before spontaneous breathing trial (SBT), SCHE, heart rate, and PaO_2_ were identified as independent predictors of weaning failure. Following propensity score matching (PSM), SCHE and heart rate remained independent predictors. Patients with SCHE levels below 3,228 u/l should undergo careful evaluation before weaning. Our findings suggest that malnutrition may be a contributing factor to weaning failure in patients.

## Introduction

In recent times, the utilization of Invasive Mechanical Ventilation(IMV) has become more prevalent, resulting in enhancing life safety for patients with acute and critical illnesses. Reports indicate that over one-third of patients admitted to the Intensive Care Unit (ICU) necessitate IMV for life-sustaining purposes (1). The weaning process is a crucial stage for mechanically ventilated patients, comprising two steps: discontinuing the ventilator and removing the tracheal intubation from the oral or nasal cavity. Therefore, it is imperative to establish an appropriate decannulation plan that involves the collaboration of all healthcare professionals. The SBT technique serves as a tool to aid clinicians in evaluating the potential risk of patient failure during the deconditioning process. This technique involves observing patients for a period of 0.5–2 h in a low-level supported spontaneous breathing mode or T-tube to determine their ability to breathe independently (2).

Several factors impact the outcomes of patient weaning, with respiratory muscle dysfunction (3), cardiac insufficiency (4), and malnutrition being the primary contributors (5). The nutritional status of patients is a significant determinant of successful weaning from mechanical ventilation. Patients receiving mechanical ventilation experience compromised nutrient absorption due to eating difficulties and disease management that disrupts gastrointestinal function. Additionally, chronic pain, inflammation, immobilization, and high catabolism render patients more susceptible to malnutrition (6). Malnourishment in patients results in heightened protein catabolism, insufficient energy provision, and unfavorable nitrogen balance, culminating in diminished respiratory muscle mass, potency, and stamina, as well as compromised ventilation. Furthermore, malnourishment impairs patients’ immune function and resistance, rendering mechanically ventilated patients more vulnerable to pulmonary infections and escalating weaning failure (7).

Given the negative impact of weaning failure on patients, the selection of an optimal time to initiate the weaning process and enhance the success rate of weaning has emerged as a critical research focus in the management of clinical use and therapeutic application of ventilators for ICU patients. The identification of appropriate weaning prediction parameters can assist clinicians in determining the appropriate timing for weaning, reducing the incidence of weaning failure, and improving patient outcomes. However, the conventional parameters utilized for weaning, such as blood oxygen saturation, brain natriuretic peptide, and shallow rapid breathing, may not provide adequate precision for predicting successful weaning due to their inherent limitations in clinical practice (8–10). Therefore, there is an urgent clinical need for predictors of offline success.

The human body generates two distinct types of cholinesterases, namely acetylcholinesterase, which is present in the brain, nerves, and red blood cells, and butyrylcholinesterase, a nonspecific cholinesterase which is present in the liver, pancreas, central nervous system, and blood. The measurement of serum butyrycholinesterase concentration is being utilized as a marker for cholinesterase levels. Prior research has established that a decreased concentration of this enzyme is linked to liver damage, inflammation, and malnourishment (11). During our regular clinical practice, we observed a potential correlation between abnormal SCHE concentrations and weaning outcomes in patients. As a result, the objective of this study was to identify predictive factors for ventilator weaning failure and to examine the relationship between low SCHE concentrations and weaning failure, while also evaluating the potential impact of low SCHE concentrations on weaning failure due to organismal malnutrition. This was accomplished by assessing the nutritional status of patients using the NUTRIC score.

## Materials and methods

### Patients and data collected

The study was conducted from January 2021 to September 2022 in the intensive care unit (ICU) of Zhejiang Hospital of Traditional Chinese Medicine, affiliated with Zhejiang University of Traditional Chinese Medicine, and retrospectively enrolled 227 adult patients treated with IMV for more than 48 h. Based on the sample content estimation formula for diagnostic tests, the minimum sample size was 90 cases. A sample size of 227 cases was included in this study, which met the minimum sample size inclusion requirement. Patients younger than 18 years of age; pregnancy; tracheotomy or other upper airway diseases; mechanical ventilation for less than 48 h; intubation before admission; abandonment before extubation; neuromuscular disease; lack of cooperation; the decision to limit active treatment and incomplete data were excluded for 148 individuals. Patients were considered ready to undergo an SBT when they fulfilled the criteria listed in the electronic Supplementary material. Demographic data, causes of IMV, the severity of illness scores, and nutritional scores were recorded at the time of ICU admission. Parameters related to blood gas analysis and ventilator mode on admission were also recorded. The duration of mechanical ventilation from the start of ventilator-assisted ventilation to the first SBT was recorded. As well as the results of vital signs, blood gas analysis, and SCHE before the start of the first SBT, and the score of the patient’s disease severity before the first SBT test.

### Study protocol

**Figure 1:**
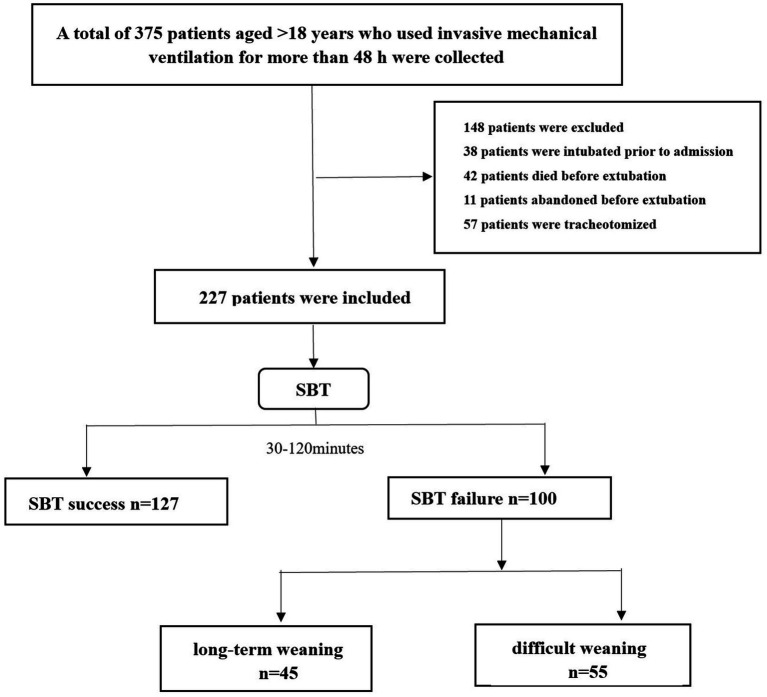
Flow chart.

This protocol has been approved by our institutional ethics committee to waive informed consent. Clinical and laboratory data were obtained for all patients at admission and before the first SBT trial (Figure 1, flowchart). SBT was performed with the patient in a semi-recumbent position, and oxygen was administered at concentrations prescribed by the attending physician through modalities such as reduced ventilator support or direct decompression. SBT lasted 30–120 min, depending on the patient’s tolerance or the physician’s decision. If the criteria for weaning failure were met, the patient was reconnected to mechanical ventilation; weaning was also considered a failure if reintubation was required within 48 h after SBT (12). According to the S2K guidelines published by the German Respiratory Society, we have divided the types of weaning into three categories (Table 1). Simple weaning group includes patients who successfully pass the first SBT and are extubated at the first attempt. Difficult weaning is defined as patients who require three SBT or up to 7 days from the first SBT to successfully wean. Prolonged weaning refers to patients with more than three SBT or >7 days of weaning after the first SBT (13, 14).

**Table 1:** Baseline characteristics of patients according to weaning outcome.

	Crude cohort			PSM model		
Characteristic	Weaning failure (*n* = 100)	Weaning success (*n* = 127)	*p*	Weaning failure (*n* = 75)	Weaning success (*n* = 75)	*p*
Age, year (median, 25th–75th percentiles)	73 (61–80)	64 (48–74)	<0.01	70 (57–76)	70 (55–79)	0.803
Male (*n*%)	74 (74%)	82 (64.6%)	0.016	52 (69.3%)	53 (70%)	0.859
Baseline (median, 25th–75th percentiles)						
APACHE II score	21 (17–26)	19 (17–23)	0.113	20 (17–25)	20 (17–24)	0.732
SOFA score	7 (5–9)	6 (5–8)	0.012	7 (5–9)	6 (5–8)	0.343
GSC score	4 (3–8)	5 (4–7)	0.450	5 (3–9)	5 (4–8)	0.786
NRS2002	5 (4–6)	5 (4–6)	0.683	5 (4–6)	5 (4–6)	0.746
Before SBT (median, 25th–75th percentiles)						
APACHE II score	18 (15–20)	16 (13–19)	0.006	17 (15–19)	17 (15–20)	0.598
SOFA score	6 (4–7)	5 (4–6)	<0.01	5 (4–6)	5 (4–6)	0.864
NUTRIC score	5 (4–6)	4 (3–5)	<0.01	5 (4–5)	4 (4–5)	0.443
IMV duration prior to weaning, days (median, 25th–75th percentiles)	9.5 (5–20.75)	6 (4–9)	<0.01	9 (4–16)	6 (4–11)	0.067
Total length of stay, days	32 (17–77)	23 (11–47)	0.001	32 (17–84)	9 (4–16)	0.174
Cause of mechanical ventilation (*n*%)						
Respiratory failure	61 (61%)	57 (44.9%)	0.016	43 (57%)	44 (59%)	0.869
Stroke	28 (28%)	42 (33.1%)	0.411	22 (29%)	52 (70%)	0.859
Septic shock	17 (17%)	13 (10.2%)	0.065	12 (16%)	7 (9%)	0.480
Pulmonary infection	50 (50%)	48 (37.8%)	0.135	38 (51%)	25 (33%)	0.624
Cardiac insufficiency	25 (25%)	12 (9.4%)	0.002	17 (23%)	11 (15%)	0.209
Hepatic insufficiency	10 (10%)	9 (7%)	0.431	8 (11%)	4 (5%)	0.229
Kidney insufficiency	21 (21%)	20 (15.7%)	0.307	15 (20%)	16 (21%)	0.840
Asthma	6 (6%)	5 (3.9%)	0.472	5 (7%)	5 (7%)	1.000
COPD	12 (12%)	8 (6.2%)	0.132	8 (11%)	6 (8%)	0.575
Traumatic injury	15 (15%)	19 (15.0%)	0.993	12 (16%)	12 (16%)	1.000

For the comparison of categorical data described by frequencies and percentages, Chi square (χ^2^) tests were performed. The Mann–Whitney U test was used for non-normally distributed data, expressed as the median (25th–75th percentile). SBT, Spontaneous breathing trial; APACHE II, Acute Physiology and Chronic Health Evaluation II; SOFA, Sequential Organ Failure Assessment; GSC, Glasgow Coma Scale; COPD, chronic obstructive pulmonary disease; IMV, Invasive Mechanical Ventilation.

### Sample collection and biomarker assays

All arterial and central venous blood gases were measured using a blood gas analyzer (ABL800, Radiometer, Copenhagen, Denmark). All arterial blood indices were measured using a fully automated five-classification blood count analyzer (BC-5180CRP, mindray, Shenzhen, China). SCHE concentrations were measured using a fully automated biochemical analyzer (AU5800, Beckman, Brea, United States).

### Statistical analysis

For the comparison of categorical data described by frequencies and percentages, Chi-square (χ^2^) tests were performed. For continuous variables, the Kolmogorov–Smirnov test was used to test the normal distribution. For normally distributed data, the t-test was used and expressed as mean ± standard deviation. For non-normally distributed data the Mann–Whitney U test was used, expressed as the median (25th–75th percentile). To identify significant predictive markers of weaning failure, factors with *p*-values less than 0.05 in the univariate analysis were included in a multifactorial logistic regression model for analysis to obtain adjusted odds ratios (OR, 95% CI). Considering the differences in baseline characteristics between the successful and failed weaning groups, propensity score (PS) matching was used to identify cohorts of patients with similar baseline characteristics. Propensity scores were estimated using a non-parsimonious multivariable logistic-regression model with the weaning outcome as the independent variable and all baseline characteristics outlined in Tables 1, 2 as covariates. A propensity score (PS) was generated for each patient based on these variables. Patients with successful weaning and those with failed weaning were matched in a 1:1 ratio without replacement, using a caliper width of 0.02. A logistic regression model was rebuilt applying the matched patients to test the effect of each factor on weaning outcome. All analyses were two-tailed, and probability values (*p* values) less than 0.05 were considered statistically significant. We used receiver operating characteristic (ROC) analysis and diagnostic calibration curves to assess the predictive power of logistic regression models, as well as to observe the sensitivity and specificity of indicators such as SCHE in predicting weaning failure and to determine the optimal cut-off values for the diagnostic markers studied. Finally, Spearman’s correlation was used for correlation analysis of the SCHE and nutritional scores, with results expressed as rho and *p* values. Statistical analysis was performed using SPSS (Statistical Package for the Social Science; SPSS Inc., Chicago, IL, United States) version 25 from Microsoft Windows or R software (version 4.2.1).

**Table 2:** Vital signs and arterial blood gases in each outcome group.

Crude cohort						
	Baseline		*p*	Before SBT		*p*
Vital signs	Weaning failure (*n* = 100)	Weaning success (*n* = 127)		Weaning failure (*n* = 100)	Weaning success (*n* = 127)	
SBT, mmHg	122.5 (106.25–139.25)	123 (111–145)	0.210	126 (112–135.75)	126 (114–144)	0.197
HR, beats/min	98 (79–114)	90 (72–108)	0.049	88 (80–96.75)	82 (69–94)	0.002
RR, breaths/min	18 (15–20)	15 (12.75–18)	0.001	18 (16–20)	17 (14–20)	0.049
Arterial blood gas						
PaO_2_, mmHg	106 (79–137)	115 (84–151)	0.101	99.5 (88.2–128.75)	110 (89–138)	0.021
PaCO_2_, mmHg	41 (35–47.4)	39 (32.3–44)	0.060	40 (35–47)	40 (35.6–45)	0.498
PH	7.38 (7.33–7.44)	7.37 (7.31–7.43)	0.404	7.441 (7.41–7.47)	7.43 (7.41–7.46)	0.222
Cholinesterase, u/l	3,867 (2,547–5345.25)	4,798 (3,363–5,745)	<0.01	3,190 (2,366–4358.25)	4,514 (3,363–5,745)	<0.01

PSM model						
	Baseline		*p*	Before SBT		*p*
Vital signs	Weaning failure (*n* = 75)	Weaning success (*n* = 75)		Weaning failure (*n* = 75)	Weaning success (*n* = 75)	
SBT, mmHg	121 (105–136)	125 (111–149)	0.045	126 (111–135)	128 (115–145)	0.122
HR, beats/min	97 (79–112)	90 (68–105)	0.014	89 (81–97)	80 (66–92)	0.001
RR, breaths/min	17 (15–20)	16 (14–19)	0.131	18 (15–20)	17 (15–20)	0.130
Arterial blood gas						
PaO_2_, mmHg	113 (82–141)	109 (81–136)	0.922	107 (83–129)	106 (87–132)	0.438
PaCO_2_, mmHg	40 (34.7–47)	40.5 (32.3–44)	0.566	41 (35–47)	40.6 (35.1–47)	0.653
PH	7.38 (7.338–7.445)	7.4 (7.32–7.44)	0.690	7.45 (7.41–7.478)	7.43 (7.41–7.46)	0.081
Cholinesterase, u/l	4,086 (2,545–5,443)	4,475 (3,752–6,005)	0.077	3,405 (2,363–4,591)	4,303 (3,286–5,549)	0.002

Continuous variables were expressed as median and interquartile range (IQR) or (mean ± standard deviation, SD). Differences were compared by Mann–Whitney U test. RR, respiratory rate; HR, heart rate; SBP, systolic blood pressure; PaCO_2_, arterial carbon dioxide tension; PaO_2_, arterial oxygen tension; FiO_2_, oxygen uptake.

## Results

### Baseline characteristics and weaning outcome

As shown in Figure 1, a total of 227 patients participated in this study. Of this total, 100 failed during weaning (94 failed SBT and 6 failed extubation after successful SBT) and 127 were eventually weaned successfully. The main causes of tracheal intubation are respiratory failure and stroke. Table 1 shows the baseline characteristics of all patients. Prior to propensity score matching, the failure group had a longer total length of stay and higher APACHE II and SOFA scores compared with those who successfully weaned; and lower GCS scores. There was a longer duration of IMV before weaning, higher APACHE II, SOFA scores, and nutrition scores, and significant differences in RR, PaO_2_, and FiO_2_ between the two groups. By using one-to-one propensity score matching (PSM), 150 patients with successful weaning were matched with 150 patients with failed weaning. After matching, there were no significant differences in *p*-values for most variables between the two groups indicating good propensity score matching. That is, there was only a small difference in baseline characteristics between the successful weaning and failure groups (Tables 1, 2).

### Multifactorial logistic regression predicts indicators of weaning failure

We used a multifactorial logistic regression model to assess the correlation between various factors and weaning events in the crude cohort and PSM model.

The findings of both the crude cohorts and the PSM model are shown in Table 3. In the crude model, before-SBT heart rate, PaO_2_, and SCHE were found to be independent predictors of weaning failure by multifactorial analysis (*p* = 0.009; *p* = 0.036; *p* = 0.006). In the propensity score model, before-SBT SCHE and heart rate remained independent predictors (*p* = 0.05; *p* = 0.006).

**Table 3:** Risk factors for weaning failure.

	Crude cohort				PSM model			
Variables	Exp (β)	95%CI		*p*	Exp (β)	95%CI		*p*
			
Age	1.002	0.998	1.007	0.373	1.011	0.981	1.042	0.490
SOFA	1.154	0.940	1.415	0.170	1.062	0.907	1.244	0.458
HR	1.007	0.992	1.022	0.344	1.005	0.988	1.023	0.549
RR	1.012	0.969	1.057	0.585	1.014	0.937	1.098	0.726
Baseline-cholinesterase	1.000	1.000	1.000	0.313	1.000	1.000	1.000	0.362
Before SBT HR	1.028	1.007	1.049	0.009	1.039	1.011	1.068	0.006
Before SBT PaO_2_	0.989	0.979	0.999	0.036	0.989	0.978	1.000	0.055
Before SBT cholinesterase	1.000	0.999	1.000	0.006	1.000	0.999	1.000	0.050
APACHE II	1.045	0.952	1.147	0.355	1.085	0.964	1.222	0.177
Before SBT Nurtic	0.891	0.531	1.494	0.661	0.545	0.304	0.978	0.042
Respiratory failure	0.624	0.327	1.188	0.624	(−)			
Cardiac insufficiency	0.467	0.188	1.158	0.151	(−)			
LOS	1.002	0.998	1.007	0.253	1.002	0.998	1.007	0.336

HR, heart rate; RR, Respiratory rate; LOS, length of stay.

**Figure 2:**
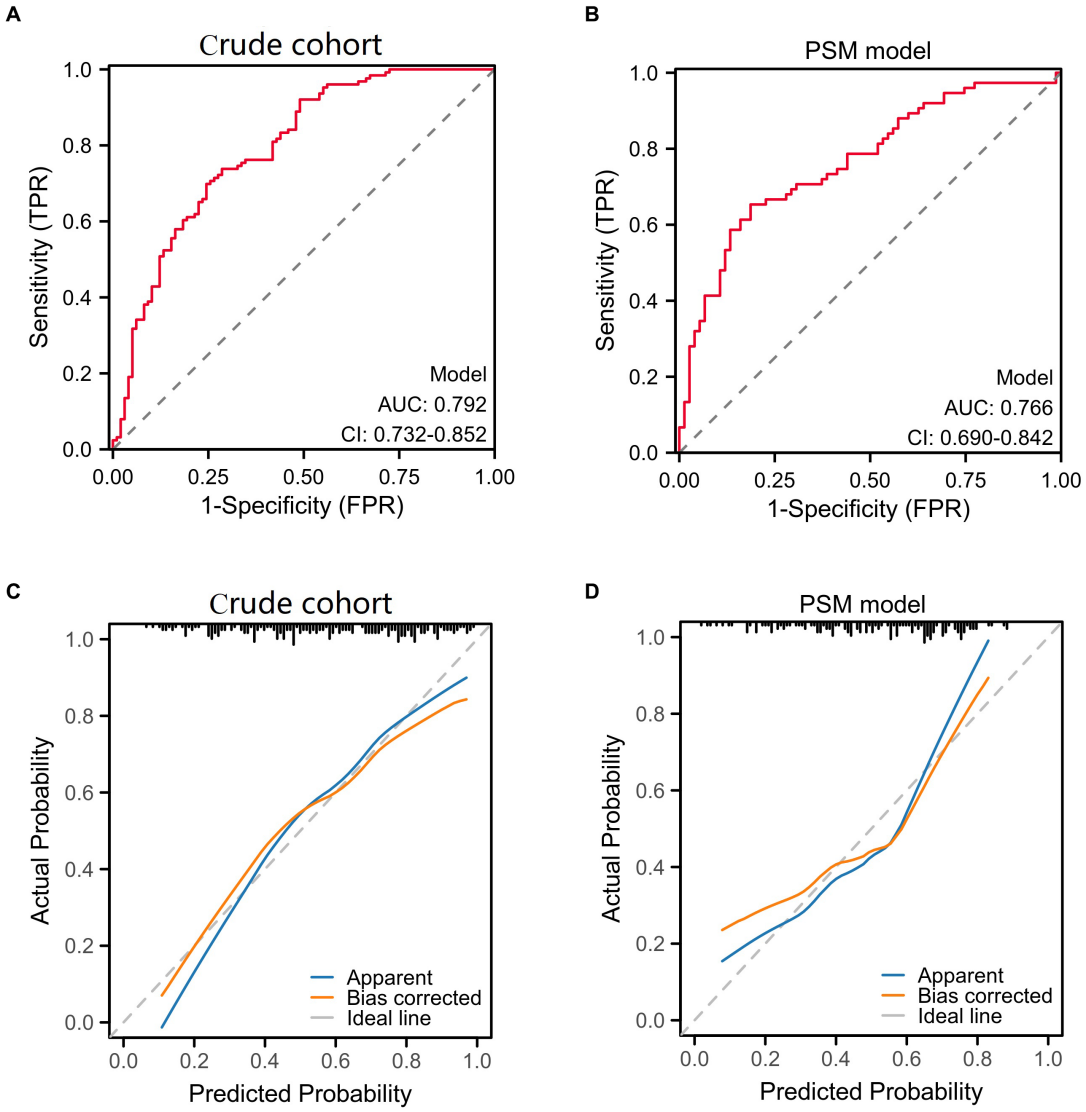
Prognostic model validation curve.

We used receiver operating characteristic (ROC) analysis and diagnostic calibration curves to assess the predictive power of logistic regression models. Figure 2 shows that the AUC for crude cohort (0.792; 95% CI 0.732–0.852) was higher than that for PSM model (0.766; 95% CI 0.690–0.842). The calibration curves for the crude cohort and PSM model showed no significant deviation from the reference line, The C-indexes were 0.792 and 0.766 for the crude cohort and PSM model indicating a relatively fair agreement between the PSM model predictions and crude cohort (Figure 2).

**Figure 3:**
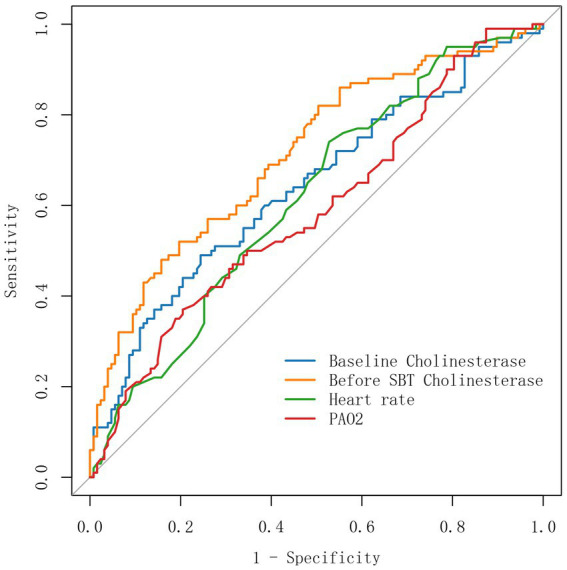
Area under the operating characteristic (AUC ROC) curve used to predict weaning failure.

The performance of baseline SCHE, SCHE before SBT, heart rate, and PaO_2_ in predicting weaning failure was evaluated by calculating the area under the subject’s working characteristic curve (Figure 3 and Table 4). The optimal thresholds for predicting weaning failure were before SBT SCHE <3,228 u/l, baseline SCHE <3,744 u/l, heart rate >80.5 beats, and PaO_2_ <87.7 mmHg. Table 4 shows that the AUC for before-SBT SCHE (0.714; 95% CI 0.647–0.782) was higher than that for baseline SCHE (0.641; 95% CI 0.568–0.715) and heart rate (0.618; 95% CI 0.545). The diagnostic accuracy before SBT SCHE was the highest.

**Table 4:** ROC curves for baseline cholinesterase, before SBT cholinesterase, heart rate, and PaO_2_.

	Cut off	AUC	AUC 95%CI		*p*	ACC	SEN	SPE	PPV	NPV	Index
Bacraseline cholinesterase	3,744	0.641	0.568	0.715	<0.01	0.639	0.490	0.756	0.613	0.653	0.246
Before SBT cholinesterase	3,228	0.714	0.647	0.782	<0.01	0.678	0.520	0.803	0.675	0.680	0.323
Heart rate	80.5	0.618	0.545	0.690	0.001	0.590	0.740	0.472	0.525	0.698	0.212
PaO_2_	87.7	0.590	0.515	0.664	0.01	0.608	0.370	0.795	0.587	0.616	0.165

ACC, Accurate value; SEN, Sensitivity; SPE, Specificity; PPV; Positive predictive value; NPV, Negative predictive value.

### Weaning difficulty and SCHE concentration

According to the patients’ weaning condition, they were divided into two types: difficult weaning and long-term weaning, and long-term weaning was more difficult and severer than difficult weaning. Therefore, we performed a subgroup analysis to explore the correlation between SCHE concentration and patients’ weaning difficulty by ordered logistic regression (Table 5). The results showed that SCHE before SBT was strongly correlated with patients’ weaning difficulty (*p* < 0.01), and so does the heart rate and PaO_2_ before SBT (*p* = 0.009, *p* = 0.008). According to the regression coefficients, SCHE and PaO_2_ were negatively correlated with weaning difficulties, and heart rate was positively correlated with weaning difficulties.

**Table 5:** Ordinal logistic regression.

	*p*	Regression coefficient	95% CI	
Before SBT SCHE	<0.01	−0.000335	−0.001	0.00
PaO_2_	0.009	−0.011	−0.019	−0.003
Heart rate	0.008	0.022	0.006	0.039

### SCHE and nutrition

Patients’ nutrition was assessed before weaning and on the day of SBT according to the NUTRIC score. Spearman correlation analysis was performed between the SCHE concentration and the NUTRIC score of patients before SBT, and the results showed that SCHE was negatively correlated with NUTRIC scores (*p* < 0.01, Table 6). Therefore, patients with lower SCHE were found to be at higher nutritional risk.

**Table 6:** Correlation of cholinesterase and NURTIC scores.

		Serum cholinesterase	NUTRIC score
Serum cholinesterase	RHO	1.000	−0.309
	*p*	–	<0.01
NUTRIC score	RHO	−0.309	1.000
	*P*	<0.01	–

Correlation analysis was performed using Spearman’s correlation and the results are shown as correlation coefficient rho and *p* value. PaO_2_, arterial oxygen tension; NUTRIC score, The Nutrition Risk in Critically ill.

## Discussion

This research aimed to investigate the relationship between SCHE and other indicators with weaning outcomes. A cohort study with a one-to-one propensity score-matching approach revealed that low SCHE was significantly associated with weaning failure following invasive mechanical ventilation. Further analysis of subgroups with varying degrees of weaning difficulty facilitated a more comprehensive understanding of the relationship between SCHE and different levels of weaning failure. Additionally, our findings indicated that low SCHE is correlated to a high nutritional risk. The crude cohort exhibited a negative correlation, which was also observed in the logistic regression model established through the PSM approach.

The state of critical illness is characterized by a significant catabolic effect, and in the event of insufficient nutritional interventions, patients are at risk of malnutrition, which can result in unfavorable clinical outcomes (15). Additionally, energy expenditure and nitrogen loss exhibit temporal variability, underscoring the importance of appropriate nutritional therapy and optimal dosing (5, 16). Malnutrition may contribute to respiratory muscle weakness and heighten the likelihood of aspiration due to dysphagia, while also impacting the patient’s cardiopulmonary exercise and weaning (17, 18).

According to previous research, SCHE levels have demonstrated efficacy as a biomarker for identifying older adults who are susceptible to muscle loss (19). Furthermore, SCHE has been shown to play a significant role in lipid metabolism (20). Consequently, SCHE levels are considered to be a comprehensive biomarker for malnutrition in theory. Reduced serum SCHE levels have been observed in various clinical contexts, including malnutrition, inflammation, and liver damage. In the current investigation, patients with low SCHE levels exhibited higher NUTRIC scores, indicating a greater nutritional risk. Prospective clinical studies have reported that nutritional support can be beneficial for patients at high nutritional risk (21, 22). Furthermore, SCHE levels have demonstrated moderate correlation with albumin levels, body mass index (BMI), and other nutritional indicators, while exhibiting weak or no correlation with liver function test results and C-gamma reactive protein levels. These findings suggest that SCHE levels are primarily influenced by malnutrition rather than inflammation or liver function (23, 24). Notably, both serum SCHE and albumin are synthesized in the liver and possess serum half-lives of 8–11 days and 12–17 days, respectively. Compared to albumin, the half-life of SCHE is shorter and more responsive, so SCHE starts to decrease earlier than albumin Consequently, SCHE may be more appropriate for dynamic nutritional status testing (25). Furthermore, In patients receiving albumin supplementation, the level of hepatic synthesis of albumin is difficult to detect, but can be replaced by monitoring the SCHE (26). The literature has also documented that SCHE stands out as the most robust prognostic indicator among various nutritional parameters (27).

This study presents an investigation into the potential utility of serum cholinesterase (SCHE) levels as a predictor of successful ventilator weaning (<3,228 u/l). The study found a negative correlation between SCHE concentration and nutritional risk, which sheds light on the role of SCHE in the occurrence of ventilator weaning failure. The findings of this study highlight the significance of SCHE levels in guiding the timing of weaning and evaluating the nutritional status of patients.

## Limitations

This study has several limitations. First, this was a single-center retrospective study with a relatively small sample and short duration, and the results of this study should be considered with caution. Second, we did not study medications that may affect SCHE, such as drugs that may impair liver function such as antibiotics. Second, certain complications, nutritional status, and anti-inflammation may affect withdrawal outcomes, but data are lacking because of the difficulty of obtaining all data in the ICU. Third, due to a lack of data, we did not assess the relevance of other more classical parameters associated with deconditioning, such as rapid shallow respiratory index or negative inspiratory force, or grip strength.

## Conclusion

In conclusion, the presence of lower serum cholinesterase (SCHE) concentrations may have a negative impact on the withdrawal of mechanical ventilation, and this decrease in SCHE levels is linked to malnutrition. It is recommended that SCHE assessment be correlated with other prognostic indicators. Additionally, tachycardia or lower arterial partial pressure of oxygen prior to weaning are positive predictors of weaning failure. Therefore, SCHE concentrations hold significant clinical value, and further large-scale prospective studies are necessary.

## Data availability statement

The raw data supporting the conclusions of this article will be made available by the authors, without undue reservation.

## Ethics statement

The Ethics Committee of Zhejiang Hospital of Traditional Chinese Medicine, affiliated with Zhejiang University of Traditional Chinese Medicine, approved the study (NO.2023-KLS-003-01). Due to the retrospective study, the Ethics Committee of Zhejiang Hospital of Traditional Chinese Medicine Affiliated with Zhejiang University of Traditional Chinese Medicine agreed to waive written informed consent and we kept the clinical data of the patients confidential. All procedures were by the Declaration of Helsinki. All personal information was encrypted in the database and was anonymous, so there was no invasion of privacy.

## Author contributions

JL and TS designed the study and completed the first draft. CM provided technical support for statistical analysis. HC, XL, and XY collected and analyzed the data. KS participated in the revision of the manuscript. DW, LW, and SL provided financial support. JL,TS, HC, CM, XL, XY, KS, LW, SL, and DW wrote the paper. All authors contributed to the article and approved the submitted version.

## Funding

This study was supported by 2022 Supported Discipline of Zhejiang Provincial Hospital of Chinese Medicine(No.2022-65); 2022 University-level Basic Scientific Research Ability Improvement Project of Zhejiang Chinese Medical University(No.2022JKJNTZ24); the National Natural Science Foundation of China (No.82104587); 2022 National Traditional Chinese Medicine Administration Project For the Traditional Chinese Medicine Rehabilitation Service Capability Improvement (No. 2021-242); Zhejiang Provincial Medical and Health Technology Program(No. 2019ZD044).

## Acknowledgments

We thank all the researchers and members of the Department of Critical Care Medicine, Pulmonary and Critical Care Medicine for their efforts.

## Conflict of interest

The authors declare that the research was conducted in the absence of any commercial or financial relationships that could be construed as a potential conflict of interest.

## Publisher’s note

All claims expressed in this article are solely those of the authors and do not necessarily represent those of their affiliated organizations, or those of the publisher, the editors and the reviewers. Any product that may be evaluated in this article, or claim that may be made by its manufacturer, is not guaranteed or endorsed by the publisher.

## Abbreviations

SCHE: Serum Cholinesterase
IMV: invasive mechanical ventilation
SBT: autonomic breathing test
ICU: Intensive Care Unit
APACHE II: Acute Physiology and Chronic Health Evaluation II
SOFA: Sequential Organ Failure Assessment
GSC: Glasgow Coma Scale
COPD: chronic obstructive pulmonary disease
RR: respiratory rate
HR: heart rate
SBP: systolic blood pressure
PaCO_2_: arterial carbon dioxide tension
PaO_2_: arterial oxygen tension
FiO_2_: oxygen uptake
LOS: length of stay
