# Supplementary material for: Serum cholinesterase as a new nutritional indicator for predicting weaning failure in patients
Source: Front Med (Lausanne). 2023 Jul 12;10:1175089. doi: 10.3389/fmed.2023.1175089 (PMC10368973; doi:10.3389/fmed.2023.1175089)
Supplement: Supplementary file 1 [file Data_Sheet_1.docx]

Weaning process

Patients selected for this study based on inclusion and exclusion criteria were identified by their treating physician's medical team (including a chief physician and three attending physicians) from 9:00 to 11:00 a.m. each day, taking into account the patient's condition and clinical experience, to determine whether the patient met the criteria for weaning assessment, including the following four components.

(1) The cause of the patient's respiratory failure requiring mechanical ventilation for respiratory support has improved or been eliminated.

(2) Good oxygenation status (general patients need to meet pH ≥ 7.25, oxygenation index ≥ 150~300mmHg, oxygen concentration ≤ 0.40, positive end-expiratory pressure ≤ 5~8cmH2O; COPD patients meet pH > 7.30, oxygen partial pressure > 50mmHg, oxygen concentration < 0.35.)

(3) Hemodynamic stability without progressive myocardial ischemia with significant hypotension (without the use or with only small doses of vasoactive drug support, such as dobutamine or dobutamine at doses <5~10ug/kg/min).

(4) The patient can breathe spontaneously.

Autonomic breathing test

Autonomic breathing test mainly includes T-tube test, continuous positive airway pressure ventilation, and low-level pressure support ventilation. 35 breaths/min; heart rate <140 beats/min or <20% change, no new arrhythmia; tidal volume during spontaneous breathing >4 ml/kg; oxygen saturation >90% then a longer (30 minutes) SBT is performed. On the contrary, if the patient exceeds the above-mentioned indexes or shows changes in mental status such as excitement, drowsiness, coma, labored breathing, paradoxical breathing, etc., the 3-minute SBT is judged to have failed, and the ventilator-assisted breathing is continued as prescribed by the physician, who sets the appropriate ventilator parameters according to the patient's condition, gives the patient adequate rest for the respiratory muscles, and continues with the offline screening test the next day. After 30 minutes of SBT, if the patient met the following indicators, SBT was judged to be successful, and the medical staff promptly prepared to remove the tracheal intubation. Arterial blood gas indexes: (oxygen concentration <0.40, oxygen saturation ≥85%~90%, partial pressure of oxygen ≥50~60mmHg, pH≥7.32, partial pressure of carbon dioxide increased ≤10mmHg); stable hemodynamic indexes (heart rate <120~140 beats/min and heart rate change <20%, systolic blood pressure <180~200mmHg and >90mmHg, blood pressure (change of blood pressure <20%, no need for vasoactive drugs); respiratory rate ≤30~35 breaths/min and respiratory rate change ≤50%).
